# Assessing the validity of ChatGPT-4o and Google Gemini Advanced when responding to frequently asked questions in endodontics

**DOI:** 10.1590/1678-7757-2025-0321

**Published:** 2025-09-29

**Authors:** Nicolás Dufey-Portilla, Ana Billik Frisman, Maximiliano Gallardo Robles, Fernando Peña-Bengoa, Consuelo Cabrera Ávila, Venkateshbabu Nagendrababu, Paul M. H. Dummer, Marc Garcia-Font, Francesc Abella Sans

**Affiliations:** 1 Universidad Andres Bello Department of Endodontics School of Dentistry Viña del Mar Chile Universidad Andres Bello Department of Endodontics, School of Dentistry, Viña del Mar, Chile.; 2 Autonomous research Viña del Mar Chile Autonomous research, Viña del Mar, Chile.; 3 Universidad Andres Bello Endodontic Specialty Program School of Dentistry Viña del Mar Chile Universidad Andres Bello, Endodontic Specialty Program, School of Dentistry, Viña del Mar, Chile.; 4 University of Sharjah College of Dental Medicine Department of Restorative Dentistry Sharjah United Arab Emirates University of Sharjah, College of Dental Medicine, Department of Restorative Dentistry, Sharjah, United Arab Emirates.; 5 Cardiff University College of Biomedical and Life Sciences School of Dentistry Cardiff United kingdom Cardiff University, College of Biomedical and Life Sciences, School of Dentistry, Cardiff, United kingdom; 6 Universitat International de Catalunya Department of Endodontics. Sant Cugat del Valles School of Dentistry Barcelona Spain Universitat International de Catalunya, School of Dentistry, Department of Endodontics. Sant Cugat del Valles, Barcelona, Spain.

**Keywords:** Artificial intelligence, ChatGPT, Endodontics, Google Gemini, Large language models

## Abstract

Artificial intelligence (AI) is transforming access to dental information via large language models (LLMs) such as ChatGPT and Google Gemini. Both models are increasingly being used in endodontics as a source of information for patients. Therefore, as developers release new versions, the validity of their responses must be continuously compared to professional consultations. Objective: This study aimed to evaluate the validity of the responses provided by the most advanced LLMs [Google Gemini Advanced (GGA) and ChatGPT-4o] to frequently asked questions (FAQs) in endodontics. Methodology: A cross-sectional analytical study was conducted in five phases. The top 20 endodontic FAQs submitted by users to chatbots and collected from Google Trends were compiled. In total, nine academically certified endodontic specialists with educational roles scored GGA and ChatGPT-4o responses to the FAQs using a five-point Likert scale. Validity was determined using high (4.5-5) and low (≥4) thresholds. The Fisher's exact test was used for comparative analysis. Results: At the low threshold, both models obtained 95% validity (95% CI: 75.1%- 99.9%; p=.05). At the high threshold, ChatGPT-4o achieved 35% (95% CI: 15.4%- 59.2%) and GGA, 40% (95% CI: 19.1%- 63.9%) validity ( *p=1* ). Conclusions: ChatGPT-4o and GGA responses showed high validity under lenient criteria that significantly decreased under stricter thresholds, limiting their reliability as a stand-alone source of information in endodontics. While AI chatbots show promise to improve patient education in endodontics, their validity limitations under rigorous evaluation highlight the need for careful professional monitoring.

## Introduction

Artificial intelligence (AI) consists of a branch of computer science aimed at simulating human intelligence by systems trained on databases and advanced algorithms to provide responses comparable to human reasoning.^1 , 2^ This constantly evolving field has developed large language models (LLMs), also known as chatbots.^3^ The key emerging capabilities of LLMs include contextual learning, instruction resolution,^4^ and conversation simulation with immediate responses.^5 , 6^

Due to their accessibility and ease of use, LLMs have introduced a new way to obtain information, raising valid concerns about the validity of the provided information and the level of trust users may place in these models. In dentistry, ChatGPT has been documented as capable of diagnosing conditions, supporting decision-making, analyzing clinical and radiographic images, and serving as an information source guiding users by various treatments, including those in endodontics.^7^ In this area, only a few studies have investigated their use as a source of information for patients based on earlier versions of these chatbots.^8 , 9^

The most frequently used LLM is the Generative Pre-trained Transformer chatbot (ChatGPT), developed by OpenAI.^10^ Its latest version, ChatGPT-4o (in which "o" stands for "omni") supports a combination of text, audio, image, and video inputs and generates supposedly high-quality responses.^11^ According to its developers, ChatGPT-4o exceeds its previous version in accuracy and contextual relevance when providing information.^12^

The Google AI LLM known as Gemini uses Google Brain transformers to process large amounts of textual data.^5^ According to its developers, its most advanced model, Google Gemini Advanced (GGA), is the first to achieve human-expert-level performance on the Massive Multitask Language Understanding test. This benchmark evaluates knowledge and reasoning across various secondary and university exams. The model achieved a score exceeding 90%.^5^

Various reports have described the validity of the information provided by LLMs.^8 , 9^ For example, Mohammad-Rahimi, et al.^9^ (2024) assessed the validity of endodontic information generated by ChatGPT-3.5 and Google Bard, concluding that ChatGPT-3.5 outperformed Google Bard. However, the chatbots in these early reports are outdated and have been superseded by updated versions that are yet to be evaluated for their validity. Therefore, this study aimed to assess the validity of responses provided by GGA and ChatGPT-4o to FAQs in endodontics that had been submitted by their users. The null hypothesis of this study postulated no statistically significant difference in the validity of responses between the two models.

## Methodology

### Ethical approval

The Ethics and Scientific Committee of Universidad Andrés Bello (Chile) reviewed and approved this cross-sectional, analytical, observational study under Resolution 71/2024.

### Study design

This is an observational, analytical, cross-sectional investigation. Its primary objective was to evaluate the validity of the responses provided by ChatGPT-4o and GGA, the two most common AI LLMs platforms in healthcare,^13^ to 20 FAQs in endodontics from users of these platforms. This study was conducted in five phases, adapting the methodology proposed by Mohammad-Rahimi, et al. ^9^ (2024).

### Phase 1: FAQs selection process

FAQs collect common queries and responses regarding a specific area. This study found and selected the 20 most essential FAQs about endodontics on GGA and ChatGPT-4o. These questions were retrieved by directly instructing both language models with the following prompt: " *As an advanced artificial intelligence model assistant, provide the 20 most FAQs users consult you about in the area of endodontics, the dental specialty dedicated to the treatment and prevention of diseases affecting the dental pulp and periradicular tissues* ".

In total, 13 of the 20 initial questions were overlapped between the two models (Q1–Q13), whereas four were exclusive to GGA (Q14–Q17) and three to ChatGPT-4o (Q18–Q20). However, regardless of their original source, all questions underwent further refinement and standardization to create a single, unified set of 20 FAQs that was then shown to both chatbots under identical conditions. Therefore, the initial origin of the questions failed to influence the validity assessment.

The inclusion criteria for question selection were as follows:

- Questions exclusively related to endodontics, including terminology, treatments, potential complications and risks, post-treatment care, and the description and meaning of endodontic pain symptoms.
- Questions endodontic specialists could answer with scientifically-based information.
- Exclusion criteria included:
- Questions unrelated to endodontics.
- Questions about treatment costs.
- Questions ignored by the chatbots.

### Phase 2: Question refinement

**Figure 1:**
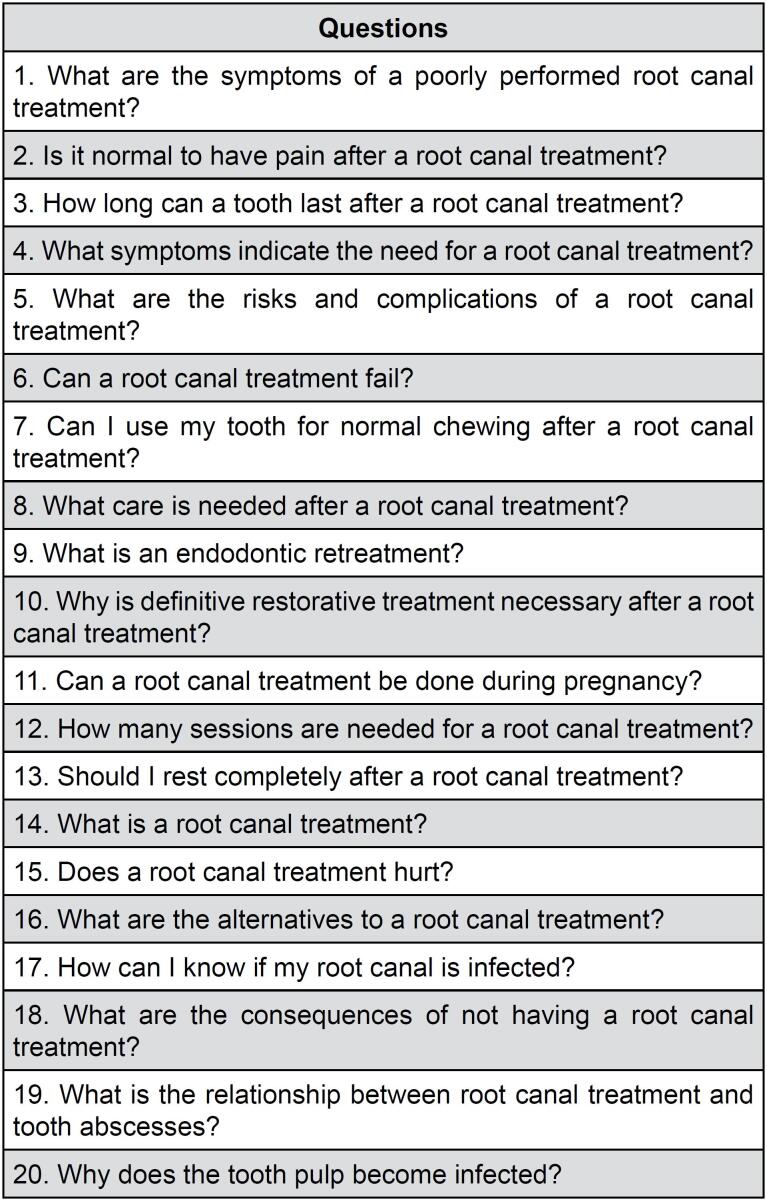
Frequently Asked Endodontic Questions derived from Google Gemini Advanced and ChatGPT-4o.

FAQ selection initially relied on ChatGPT-4o and GGA to generate a representative set of FAQs. However, this approach introduces potential bias as chatbots formulate questions based on their training data rather than actual patient concerns. To address this limitation, chatbot-generated FAQs were cross-referenced with publicly available patient inquiries from Google Trends data on common endodontic-related searches. Then, two endodontic specialists with educational roles (N.D. and C.C.) reviewed and refined the 20 questions for clarity and comprehension, ensuring that the chatbots would generate clear and coherent responses without altering the content or meaning of the questions. ( Figure 1 ).

### Phase 3: Question formulation and response retrieval

The refined 20 questions were individually and simultaneously queried to each chatbot on the same date and time (October 26, 2024, 5:40 PM) using the following prompt:

*"Assume the role of an endodontic specialist and provide a precise answer to the following question."*

Overall, two separate Google Chrome tabs were used, one for GGA and another for ChatGPT-4o. To minimize the potential influence of previous interactions or model learning memory, both chatbots were accessed on newly created Google accounts with no prior usage history. Each question was submitted in a clean browser session, the cache and cookies of which were cleared before the interaction. They were accessed via https://gemini.google.com/app and https://chatgpt.com , respectively. Responses were documented on Google Docs, and each webpage was refreshed after every interaction. Only the first answer from each chatbot was collected and evaluated. This approach was intended to simulate a real user-chatbot interaction, capturing the initial response as it would typically appear to a patient or clinician seeking immediate information.

### Phase 4: Response evaluation by endodontists

Responses from both chatbots were independently evaluated by nine academically certified endodontic specialists with educational roles at Universidad Andrés Bello (Viña del Mar, Chile). Their expertise encompassed a wide range of clinical experience spanning from five to 20 years. Additionally, all evaluators were affiliated with academic institutions and had prior experience in endodontic research and education. The sample size was determined using a finite population formula with a 95% confidence interval and a 5% margin of error.

Participants signed informed consent forms, ensuring their voluntary and anonymous participation. Evaluation employed a five-point Likert scale and a modified Global Quality Score1, assigning numerical ratings based on context and content accuracy:

- 5 (Strongly Agree): The response is correct and complete.
- 4 (Agree): The response is correct but missing minor details or contains minor errors.
- 3 (Neutral): The response is partially correct, with most details being incorrect, missing, or irrelevant.
- 2 (Disagree): The response is incorrect but contains some correct elements.
- 1 (Strongly Disagree): The response is entirely incorrect or irrelevant.

Experts received anonymized forms for evaluation (Form A: ChatGPT-4o responses; Form B: GGA responses) to ensure unbiased assessments.

### Phase 5: Statistical analysis

Averages of the nine evaluations were calculated for each question to categorize responses as valid or invalid under two thresholds—as adapted from the methodology proposed by Mohammad-Rahimi, et al.^9^ (2024)

- High Threshold: Valid responses scored from 4.5 to 5.
- Low Threshold: Valid responses scored ≥4.

These cut-off averages were selected based on prior research assessing AI-generated endodontic content, ensuring consistency with the literature.^9^

The Fisher's exact test, with a 0.05 statistical significance level, was used to compare the validity of chatbot responses. The Fleiss’ Kappa coefficient measured interrater agreement between evaluators. Analyses were conducted on RStudio, version 2024.04.2+764 (Posit PBC, Inc., Boston, MA, USA).

## Results

### Summary of responses

Both chatbots successfully answered all 20 questions, yielding 40 answers for evaluation. GGA generated more concise responses, with a mean word count of 199 (range: 70–282), whereas ChatGPT-4o produced more elaborate answers, averaging 242 words per response (range: 145–438).

Regarding response summarization, **GGA included summaries in three out of 20 responses (15%),** whereas **ChatGPT-4o included summaries in 13 out of 20 responses (65%)** . Both models also occasionally recommended that users consult a dental professional. **GGA made this suggestion in 11 instances (55%)** , when compared to the **eight instances for ChatGPT-4o (40%).**

### Expert evaluations

**Figure 2:**
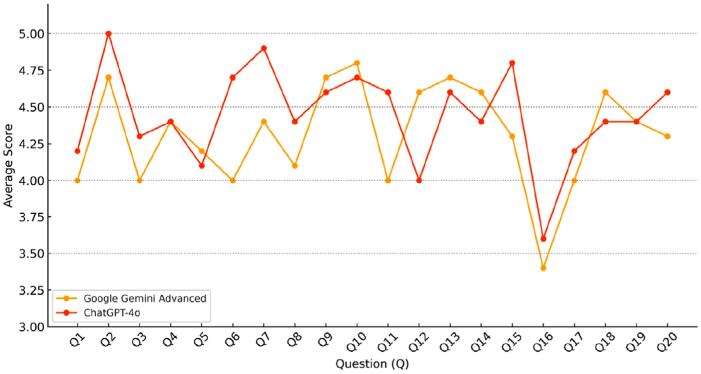
Average scores assigned by nine endodontic specialists to the responses of ChatGPT-4o and Google Gemini Advanced (GGA) to 20 questions (Q). ChatGPT-4o outperformed GGA in Q6, Q7, Q11, Q15, and Q20, whereas GGA achieved higher average scores in Q12, Q13, Q14, and Q18. Notably, only the responses to Q16 were deemed invalid (average score <4) by both models.

Overall, nine endodontic specialists independently assessed all responses. Using Fleiss’ Kappa coefficient to determine interrater reliability, **a value of 0.86 (95% CI: 0.83–0.89)** was obtained, indicating a high level of agreement between evaluators. The average scores assigned to each chatbot response ranged from 3.4 to 5.0 ( Figure 2 ).

A qualitative review of the responses with average scores below the validity threshold (<4) showed that only one question, Q16: "What are the alternatives to a root canal treatment?", was considered invalid for both chatbots (GGA: 3.4; ChatGPT-4o: 3.6). Although the responses included no false or contraindicated information, evaluators noted a lack of clinical depth, insufficient contextualization of options, and inconsistencies in how alternatives were framed. Shortcomings primarily involved overgeneralizations, limited explanation of indications or limitations for each option, and vague language that could mislead patients without professional guidance.

### High-threshold validity (4.5-5)

**Figure 3:**
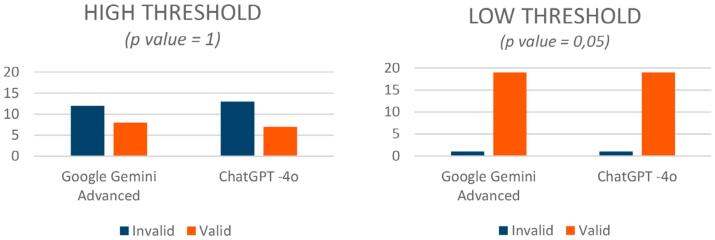
High- and low-threshold validity test comparison of chatbot responses based on average evaluation scores. The high-threshold validity required a minimum score of 4.5, whereas the low-threshold validity was set at ≥4 on a five-point Likert scale. No significant differences occurred between the two chatbots at either threshold (p≥0.05).

When applying the high-validity threshold, GGA achieved 40% validity (8 out of 20 responses; 95% CI: 19.1%–63.9%), whereas ChatGPT-4o, 35% (7 out of 20 responses; 95% CI: 15.4%–59.2%). Despite the marginally higher score of GGA, the difference was statistically insignificant ( *p=1* ) ( Figure 3 ).

### Low-threshold validity (≥4)

Under the more lenient validity threshold, both chatbots performed similarly, showing high levels of validity. GGA and ChatGPT-4o each achieved 95% validity (19 out of 20 responses; 95% CI: 75.1%–99.9%), with no statistically significant difference between the models ( *p=0.05* ) ( Figure 3 ).

## Discussion

AI-based LLMs such as Google Gemini and ChatGPT are gaining popularity as information sources.^14 , 15^ These systems immediately respond patients’ inquiries. However, the need for greater transparency regarding the data sources to train these systems raises concerns about the validity of their answers, especially in endodontics, in which information accuracy and reliability are crucial for patient safety.^15^

Recent years have witnessed a rapid expansion in the use of LLMs. While they have proven helpful in less complex tasks,^5 , 9^ healthcare providers must carefully monitor their use as sources of patient information to ensure its validity. Recent studies have highlighted that, despite their advanced capabilities, chatbots such as ChatGPT-4 have limitations, particularly in resolving clinical problems.^16 , 17^ The use of these models in endodontics has also been reported to provide variable response validity,^8 , 9 , 14^ underscoring the need for ongoing and updated evaluations of their latest versions.

Although the use of chatbots to interpret endodontic symptoms and guide treatment has significantly grown,^15^ the questions they generate may reflect only a subset of users, introducing selection bias.^18^ This underscores the importance of evaluating the validity of responses from the latest versions of LLMs, such as GGA and ChatGPT-4o, particularly in the context of FAQs in endodontics. These questions represent common concerns and thus could significantly impact patients’ clinical preferences, decision-making, and interaction with dentists.

Based on the statistical analyses in this study, its null hypothesis—that response validity would show no significant differences between the two models—was not rejected. While GGA and ChatGPT-4o had slight differences in performance, these differences were statistically insignificant at either the high or low threshold levels. Both tests assessed the validity of responses from these models at different levels of complexity. The high-threshold analysis indicated that GGA and ChatGPT-4o had low validity rates of 40 and 35%, respectively, with no significant difference between them. This suggests that, under stricter evaluation conditions, neither model reached the validity level of a human expert, underscoring the need to improve both systems for more accurate and reliable answers under rigorous criteria. Conversely, in the low-threshold analysis, both GGA and ChatGPT-4o achieved 95% validity, indicating stronger performance under less demanding conditions. This variation underscores the importance of interpreting chatbot validity regarding the strictness of the evaluation framework. Additionally, the low validity under stringent conditions raises concerns about the suitability of these chatbots in critical scenarios, such as guiding patients on oral health issues, prevention, and treatment.

Similar studies in endodontics on earlier versions of these models have reported significant differences in the validity of their responses. Johnson, et al.^19^ (2024) evaluated the validity of various chatbots in dental trauma, finding that Claude AI achieved superior results (80% under a low threshold and 55% under a high threshold) than Google Gemini and ChatGPT-3.5. On the other hand, Mohammad-Rahimi, et al.^9^ (2024) investigated the validity of chatbots as sources of information in endodontics. Their study reported that ChatGPT-3.5 showed higher validity (95%) under a low threshold than Google Bard and Bing (85 and 75%, respectively) and a 60% performance under a high threshold, significantly outperforming the latter under more demanding evaluative contexts. These findings highlight the importance of continuously assessing these models as patient information sources since developers constantly update their versions.^14^ Only recently has the performance of GGA and ChatGPT-4o been examined in dentistry, notably by Sismanoglu and Capan,^20^ who evaluated the models on the Turkish Dental Specialization Exam. Although both models performed comparably to human candidates overall, they showed limitations in specialty areas such as endodontics, in which the complexity of clinical reasoning and diagnostic detail posed consistent challenges. These findings are in line with our observations regarding the tendency of both models to oversimplify or generalize responses to endodontic FAQs.

In this study, both GGA and ChatGPT-4o showed variable response content, which may be attributed to the training methodologies in each model. ChatGPT-4o responses tended to be more extensive and detailed, whereas those of GGA were more concise and summarized. This aspect is particularly relevant as research has showed that the presentation of information significantly influences users’ perceptions of utility and satisfaction, particularly in healthcare contexts.^21 , 22^ The greater length and depth of ChatGPT-4o responses might be perceived as more comprehensive, potentially increasing users’ trust in its validity and applicability. Conversely, the more concise responses from GGA may facilitate more rapid interpretation but limit the thorough understanding of the topic, potentially leading to misconceptions and confusion.^23^

The findings of this study suggest that while GGA and ChatGPT-4o show potential utility in responding to FAQs in endodontics, further optimization of their underlying algorithms is necessary. This would ensure that the data sources used for their training are reliable and pertinent to endodontics, enabling them to provide more accurate responses in challenging contexts, as evinced by the high-threshold test results in this study. It is crucial for healthcare providers to continuously evaluate and monitor LLMs to ensure that their responses remain not only updated but also compliant with the required clinical and educational standards,^24 , 25^ thereby improving their utility and validity with each update.

AI chatbots, such as ChatGPT, have shown potential in various healthcare settings, including endodontics, by providing patient education and support.^26 , 27^ However, their use in clinical settings must be carefully considered due to potential risks and limitations. AI chatbots can sometimes provide inaccurate or incomplete information, with significant variability based on question difficulty.^8^ This highlights the risk of misinformation that could lead to inappropriate patient decisions. There also exists the concern that patients may over-rely on AI chatbots for medical advice, potentially bypassing professional consultations. This could lead to mismanagement of conditions if healthcare providers fail to corroborate the chatbot advice.^26^ While AI can generate generic patient information, it may lack the ability to tailor advice to individual patient needs and circumstances, which is critical in clinical settings.^28^

AI chatbots offer several advantages over traditional patient education methods, such as patient information leaflets; AI chatbots can provide immediate responses and are accessible 24/7, which can improve patient engagement and education;^29^ chatbot-generated materials often have a more positive emotional tone than traditional patient information leaflets, which can enhance patient receptivity and engagement.^28^ AI chatbots can also provide consistent information, reducing variability in patient education.^8^ However, traditional methods still have certain advantages: traditional patient information leaflets generally have higher readability scores (making them easier for patients to understand), and traditional methods involve direct interaction with healthcare providers, which can provide reassurance and enable personalized advice.^27 , 29^

Some relevant limitations of this study must be considered. Firstly, while the advanced chatbots GGA and ChatGPT-4o offer initial free and limited access, their unlimited use is linked to a subscription fee, which could restrict patient access to these chatbots. However, as with previous paid versions, once updated by developers, they typically become free and unlimited for general use, replacing the earlier free versions, which suggests their widespread use in the future. Secondly, a limitation of this study is the potential bias introduced by relying on AI chatbots (ChatGPT-4o and GGA) for the initial selection of FAQs. Since chatbots generate questions based on their training data rather than real-world patient concerns, this approach may not fully capture the most relevant inquiries from actual patients. To address this limitation, future studies should consider using patient surveys or clinical data to ensure a more representative set of FAQs and further validate the selection process. Furthermore, this study evaluated responses generated at a specific moment, meaning future updates to these LLMs will likely alter the responses obtained. The absence of longitudinal evaluations prevents the determination of whether the current results will remain consistent in future versions. Additionally, the study did not evaluate the reliability of chatbot responses, such as answer consistency over multiple sessions or users, which could provide more insight into the robustness of these models. This was a conscious choice due to the cross-sectional design that simulated a single real-world interaction. Nevertheless, we recognize this as a limitation and suggest that future research include reliability assessments to better understand the performance of LLMs in dynamic clinical settings.

Finally, based on this study's results, both models showed low response validity under strict evaluation criteria. This could reflect incomplete or inadequate answers, limiting their utility as information sources for patients in the endodontic field and creating a potential conflict with dentists. Given that chatbot validity decreased under stricter evaluation criteria, it is essential to contextualize their role in clinical practice. Rather than being portrayed solely as promising tools, their potential risks, including misinformation and patient overreliance, must also be acknowledged. A cautious approach is recommended, integrating AI-driven information with professional supervision to optimize patient education and decision-making. Future studies should explore the impact of these chatbots as information sources, particularly in areas where lower validity has been observed in more rigorous contexts. Additionally, they should evaluate user satisfaction with the information provided.

## Conclusions

GGA and ChatGPT-4o responses to FAQs in endodontics showed high validity under lenient criteria. However, their accuracy significantly decreased under stricter conditions. These findings highlight the limitations of AI in clinical practice. While AI chatbots offer potential benefits in patient education, they should complement professional expertise to minimize the risks associated with misinformation and patient overreliance.

Data availability

All data generated or analyzed during this study are included in this published article
